# Research progress on the pharmacological effects of berberine targeting mitochondria

**DOI:** 10.3389/fendo.2022.982145

**Published:** 2022-08-11

**Authors:** Xinyi Fang, Haoran Wu, Jiahua Wei, Runyu Miao, Yanjiao Zhang, Jiaxing Tian

**Affiliations:** ^1^ Institute of Metabolic Diseases, Guang’anmen Hospital, China Academy of Chinese Medical Sciences, Beijing, China; ^2^ Graduate College, Beijing University of Chinese Medicine, Beijing, China; ^3^ Graduate College, Changchun University of Chinese Medicine, Changchun, China

**Keywords:** berberine, mitochondria, pharmacological action, glycolipid metabolism disorder, tumor, cardiovascular diseases

## Abstract

Berberine is a natural active ingredient extracted from the rhizome of *Rhizoma Coptidis*, which interacts with multiple intracellular targets and exhibits a wide range of pharmacological activities. Previous studies have preliminarily confirmed that the regulation of mitochondrial activity is related to various pharmacological actions of berberine, such as regulating blood sugar and lipid and inhibiting tumor progression. However, the mechanism of berberine’s regulation of mitochondrial activity remains to be further studied. This paper summarizes the molecular mechanism of the mitochondrial quality control system and briefly reviews the targets of berberine in regulating mitochondrial activity. It is proposed that berberine mainly regulates glycolipid metabolism by regulating mitochondrial respiratory chain function, promotes tumor cell apoptosis by regulating mitochondrial apoptosis pathway, and protects cardiac function by promoting mitophagy to alleviate mitochondrial dysfunction. It reveals the mechanism of berberine’s pharmacological effects from the perspective of mitochondria and provides a scientific basis for the application of berberine in the clinical treatment of diseases.

## Introduction

Recent studies have found that berberine, an active ingredient from traditional Chinese medicine, has various medicinal effects. Berberine is not only irreplaceable for its dominance of antibacterial, anti-inflammatory and antiviral in traditional pharmacological research, but also has become a current research hotspot because of its remarkable curative effect in the treatment of tumors, diabetes, cardiovascular diseases, and digestive system disease. Although the pharmacological effects of berberine have been confirmed in clinical patients and animal models in the past decade, its specific targets and mechanism of action on the body are still unclear, which limits its further wide application and promotion in clinical practice. Mitochondria are organelles that exist in most cells and are involved in maintaining the stability of the internal environment by producing reactive oxygen species (ROS) and regulating cellular metabolism. A large number of studies have shown that mitochondria are one of the important targets for berberine to exert its pharmacological effects, but its specific mechanism needs to be further studied. Based on this, this paper intends to systematically review the research progress of berberine in improving glycolipid metabolism disorder, anti-tumor, and treating cardiovascular disease by regulating mitochondria, in order to provide a reference for elucidating the pharmacological mechanism of action of berberine.

## The pharmacokinetics and toxicology of berberine

Berberine is a natural isoquinoline alkaloid with yellow needle-like crystals in appearance, mostly in stable forms of hydrochloride and sulfate (1). Modern pharmacological research showed that berberine has inhibitory effects on a variety of gram-positive and gram-negative bacteria, and its mechanism of action may be to selectively inhibit bacterial RNA transcription, protein/lipid biosynthesis, and glycolysis. Berberine is mainly used to treat bacterial infectious diseases such as bacillary dysentery, gastroenteritis and carbuncle (2). With in-depth research, berberine has been found to have other extensive pharmacological actions, such as anti-oxidation, blood sugar and blood lipid regulation, anti-tumor, treatment of cardiovascular disease, etc. These new findings expand the range of applications of berberine (3, 4).

### The source of berberine

Berberine mainly exists in *Ranunculaceae*, *Berberaceae*, *Papaveraceae*, *Rutaceae*, *Fangchiaceae*, *Rhamnaceae* and other plants, with the highest content in *Rhizoma Coptidis* and *Phellodendron*. It can be extracted by acid water extraction, alkaline water extraction, ethanol extraction, liquid membrane method, two-phase extraction, supercritical fluid extraction, etc. Plant extraction and isolation, tissue culture, biological fermentation and chemical synthesis are the main ways to obtain berberine. Among them, chemical synthesis is the most important and direct source. At present, the generally accepted mainstream pathway of berberine biosynthesis is L-tyrosine as the starting material, which is converted into dopamine and 4-hydroxyphenylacetaldehyde, respectively, and then the skeleton is synthesized by biological enzymes such as synthase and transferase, and finally converted into natural berberine after skeleton modification.

### Pharmacokinetic study of berberine

The bioavailability of berberine is closely related to the route of administration. According to the experimental data, the bioavailability of berberine decreases in the following order of administration routes: intravenous injection, intraperitoneal injection, intragastric oral administration (5). Oral administration of berberine has the characteristics of difficult absorption, rapid metabolism and low blood concentration. Berberine has poor intestinal absorption and low bioavailability after oral administration. The study (6) found that the peak plasma concentration (Cmax) of berberine after oral administration of 40mg•kg^-1^ in rats was only 10μg•L^-1^. There are two main reasons for the low bioavailability of berberine. One is that berberine is a quaternary ammonium base with a lipid-water partition coefficient of -1.5. Drugs containing quaternary ammonium groups in the structure have strong hydrophilicity and low ability to penetrate cell membranes, which limit the transmembrane transport and intestinal absorption of drugs (7). Second, berberine is a substrate of P-glycoprotein (P-gp) (8). The P-glycoprotein is located on the cell membrane and acts as a drug efflux pump, and its transport leads to the secretion of absorbed berberine back into the intestine. In terms of clinical medication, although intravenous injection can increase the drug concentration in the blood, clinical trials found that it will cause serious side effects (e.g., drop in blood pressure and respiratory arrest). Thus, oral administration is the main clinical berberine administration method (9).

The uptake and distribution of berberine in tissues are very important for its biological activity. Studies have shown that berberine is widely distributed in the body after rapid absorption, and the liver is the main organ for the distribution of berberine. Tan et al. (10) deeply studied the organ distribution of berberine in rats, and the results showed that berberine (200mg•kg^-1^, orally) was rapidly distributed in major organs, such as the liver, kidney, muscle, lung, brain, heart, pancreas and fat. Ma et al. (11) determined the dose-related tissue concentration of the *Rhizoma Coptidis* alkaloids in mice using high-performance liquid chromatography with ultraviolet detection. The research indicated that four *Rhizoma Coptidis* alkaloids were detected in the brain, heart, and lung tissues of mice that received the oral total extract of *Rhizoma Coptidis*. Berberine can also penetrate the blood-brain barrier (12). Liu et al. (13) administered berberine to rats by gavage and found that the distribution of berberine in the liver was 70 times the exposure in the blood. Berberine and its metabolites are widely distributed in tissues and remain relatively stable, which may be the reason they are still active in the body even at low blood concentrations, and can still exert good antibacterial, anti-inflammatory, and hypoglycemic effects.

The main site of berberine metabolism in the body is the liver, which can be rapidly metabolized by a variety of P450 enzymes in the liver. The main metabolic pathway of phase I is methylation and demethylation, and the phase II metabolic pathway is glucuronidation and sulfation. At the same time, after berberine enters the body *via* oral administration, it will inevitably come in contact with gut microbiota. Gut microbiota can produce a variety of enzymes, such as glycosidase, nitroreductase, etc. Biotransformation of gut microbiota regulates berberine absorption and metabolism *in vivo*. Studies have shown that nitroreductase produced by gut microbiota is an important biological factor that positively regulates the intestinal absorption and metabolism of berberine. Berberine, which is not easily absorbed by the gut, can be converted into dihydroberberine by the nitroreductase produced by gut microbiota, and then the dihydroberberine enters the intestinal wall, where it is oxidized to berberine in the intestinal wall tissue, finally enters the blood circulation (14). In addition, the gut microbiota also plays an important role in the enterohepatic circulation of berberine metabolites. Therefore, both liver and gut microbiota are involved in the metabolism of berberine *in vivo*.

Berberine is mainly excreted in the urine and bile in animals, with less excretion in the stool. There is still a lack of human sample studies on the excretion kinetics of berberine. MA et al (15) studied the excretion kinetics of berberine after oral administration in rats and found that berberine was mainly excreted in the form of phase I metabolites in bile and urine.

### Toxicology study of berberine

The toxic effects of berberine are usually expressed as median lethal dose (LD_50_), which varies with the biological species and route of administration. Berberine acute toxicity test showed that the dose of dead mice after intragastric administration was 83.2 g•kg^-1^, while the LD_50_ after intravenous administration was 9.04 mg•kg^-1^. It can be seen that the bioavailability of oral administration is significantly different from that of intravenous administration. It is worth noting that no matter how different the doses of berberine administered by different routes are, the blood concentration of berberine at the time of mouse death is not different, about 0.5-0.7 mg•L^-1^. It is suggested that the toxicity of berberine is affected by its blood concentration (5). Emerging studies have shown that berberine is almost safe at conventional doses, with a relatively low incidence of adverse reactions, such as gastrointestinal discomfort, and transient increases in plasma bilirubin levels (16). Although berberine is relatively safe, it should be used with caution in specific circumstances to avoid adverse reactions. For example, A mouse study found that administration of berberine (5 mg/kg) could induce skeletal muscle atrophy *via* increasing atrogin-1 expression (17), whether this effect extends to humans is not currently known. Berberine can be toxic to nerve cells by affecting the mitochondrial respiratory chain and N-methyl-D-aspartic acid receptors (18). Berberine replaces bilirubin in binding to albumin, suggesting that the use of the herb and other traditional Chinese medicines containing a high proportion of berberine is best avoided in jaundiced neonates and pregnant women (19). Berberine interacts with macrolides to inhibit human ether-a-go-go-related gene (hERG) channels, leading to arrhythmias (20). Berberine in combination with statins increases cardiotoxicity by inhibiting CYP3A4 and hERG potassium channels (21).

## Research progress on the regulation mechanism of mitochondrial activity

**Figure 1:**
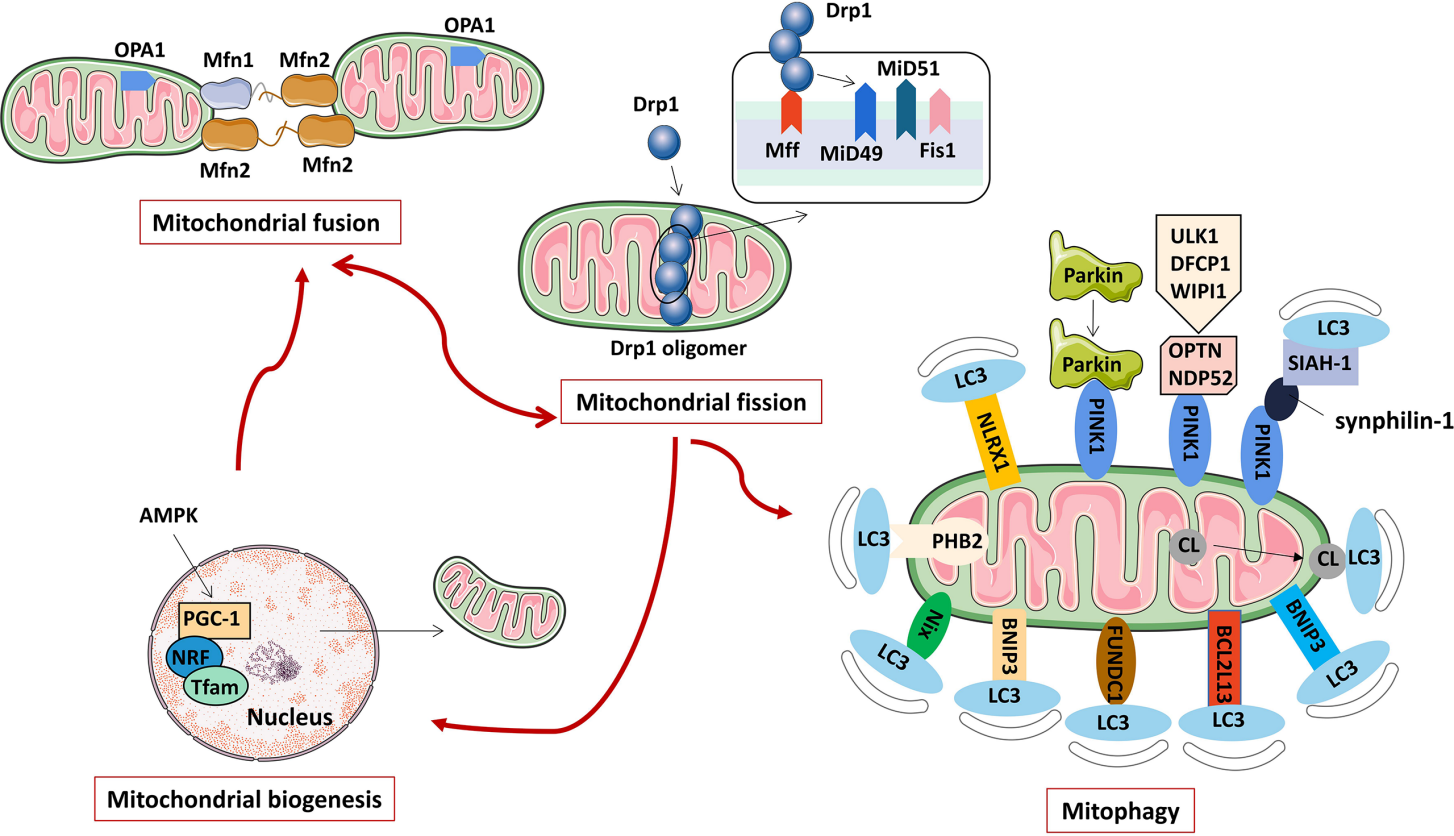
The regulation mechanism of mitochondrial activity (including mitochondrial fusion, fission, biogenesis, and mitophagy).

Mitochondria are an important organelle ubiquitous in eukaryotic cells. They are mainly divided into the outer membrane, intermembrane space, inner membrane and matrix. Different enzymes and biological factors are distributed in different spaces. For example, there are anti-apoptotic Bcl-2 family proteins and ion channel proteins on the outer membrane, cytochrome C, apoptosis-inducing factor and procaspase 2, 3, and 9 are distributed in the intermembrane space, the inner membrane is the aggregation site of the complexes that make up the mitochondrial respiratory chain complex enzymes, the tricarboxylic acid cycle-related enzymes and mitochondrial genome are distributed in the matrix. The difference in the permeability of the inner and outer mitochondrial membranes creates a trans-mitochondrial membrane potential that maintains mitochondrial integrity and performs its normal function. Mitochondria are important places for cells to perform aerobic respiration and oxidative phosphorylation to synthesize ATP. Furthermore, mitochondria are also involved in various physiological processes such as cell proliferation, differentiation, signal transduction, innate immune regulation, autophagy, and apoptosis (22). As a highly dynamic organelle, mitochondria are precisely regulated by a variety of proteins for mitochondrial fusion, fission, biosynthesis and clearance (mainly mitophagy) to ensure that mitochondria function effectively, adapt to the energy metabolism of cells, and maintain cells in various physiological activity (**Figure 1**).

### Regulatory mechanisms of mitochondrial dynamics

Mitochondrial dynamics refers to the continuous fission and fusion of mitochondria to control mitochondrial morphology, provide energy for cells, and regulate processes such as autophagy, calcium homeostasis, innate immunity, signal transduction, and apoptosis (23). Mitochondrial fission is a proliferative process that produces one or more daughter mitochondria, which are required for the formation of new mitochondria. Mitochondrial fission not only increases the number of mitochondria, but also separates damaged mitochondria from the mitochondrial network and promotes autophagy of damaged mitochondria (24). Mitochondrial fusion is a phenomenon in which two adjacent mitochondrial outer and inner membranes fuse to form a mitochondrion with a fibrous extension and a network-like structure. Mitochondrial fusion is important for maintaining normal mitochondrial function. When mitochondria are slightly damaged, mitochondria fuse, which can maintain the stability of the mitochondrial structure, allow material exchange between mitochondria, and repair their functional defects. The dynamic balance of mitochondrial fission and fusion is crucial to maintaining the quality and function of mitochondria, and is an important basis for ensuring the normal activities of cells. Enhanced or weakened mitochondrial fusion/fission protein functions can disrupt the mitochondrial dynamic balance, which may lead to oxidative stress, mitochondrial dysfunction and metabolic changes, and ultimately promote the occurrence of related diseases.

Mitochondrial fission is mainly regulated by mitochondrial dynamin-related protein1 (Drp1) and Drp1 receptor proteins located in the inner or outer mitochondrial membrane, such as mitochondrial fission factor (Mff), mitochondrial fission protein 1 (Fis1), mitochondrial dynamics proteins of 49 kDa (MiD49) and mitochondrial dynamics proteins of 51 kDa (MiD51) (25). Drp1 is a protein necessary for mitochondrial fission. It is generally located in the cytoplasm under physiological conditions. It is mainly composed of an N-terminal GTPase domain, a helical middle domain and a C-terminal GTPase effector domain (26). Under the stimulation of various mitochondrial fission factors, Drp1 is recruited to mitochondria by its receptor protein, oligomerization occurs, and multiple Drp1 assemble into cyclic polymers around mitochondria. Under the action of GTPase hydrolysis, Drp1 cyclic polymers shrink, which ruptures the inner and outer mitochondrial membranes, eventually leading to mitochondrial fission (27). After mitochondrial fission, Drp1 can revert to monomers and re-enter the cytoplasm for reuse (28).

Mitochondrial fusion includes three steps, mitochondrial tethering, mitochondrial outer membrane fusion, and mitochondrial inner membrane fusion (29). In mammals, it is mainly regulated by mitofusin 1 (Mfn1), mitofusin 2 (Mfn2) and optic atrophy 1 (OPA1) protein (30). Among them, Mfn1 and Mfn2 mainly mediate mitochondrial outer membrane fusion, and OPA1 mainly participates in the mitochondrial inner membrane fusion process. Mfn is widely expressed on the outer mitochondrial membrane, and the interaction between Mfn1 and Mfn2 undergoes cis dimerization to form Mfn1/Mfn2 homodimers or Mfn1-Mfn2 heterodimers, which promote the occurrence of adjacent mitochondrial outer membranes tethering and subsequent mitochondrial outer membrane fusion (31, 32). Next, OPA1, which localizes to the inner mitochondrial membrane, mediates mitochondrial inner membrane fusion and cristae remodeling (33). Studies have shown that OPA1 induces mitochondrial inner membrane fusion in a manner dependent on Mfn1, but not Mfn2, suggesting that there may be information transmission between the inner and outer membranes during mitochondrial fusion, and there may be an interaction between the outer membrane protein Mfn1 and the inner membrane protein OPA1 (34). However, the mechanism of OPA1-induced mitochondrial inner membrane fusion has not been fully revealed, and more in-depth studies are still needed.

### Regulatory mechanism of mitophagy

Mitophagy refers to the depolarization of mitochondria in cells under stress such as ROS, nutrient deficiency, and cell aging. The damaged mitochondria are specifically encapsulated into autophagosomes and fused with lysosomes to complete the process of degradation of damaged mitochondria, thereby maintaining a stable intracellular environment (35). Mitophagy not only removes damaged mitochondria, but also degrades normal mitochondria for survival when cells are in a harsh environment (36). Mitophagy is a type of selective autophagy that controls the quantity and quality of mitochondria and maintains the normal function of the mitochondrial network. Abnormal mitophagy can cause many pathological changes, which can lead to Alzheimer’s disease, Parkinson’s disease, heart failure, tumors and other diseases.

Mitophagy in mammalian cells mainly includes the ubiquitination degradation pathway mediated by PTEN-induced putative kinase protein 1 (PINK1) and the receptor recognition pathway mediated by different proteins such as Nip3-like protein X (NIX), Bcl-2/adenovirus E1B 19 kDa interacting protein 3 (BNIP3), FUN14 domain-con-taining protein 1 (FUNDC1), FK506 binding protein 8 (FKBP8), Bcl-2-like protein 13 (Bcl2L13), nucleotide-binding domain and leucine-rich-repeat-containing proteins X1 (NLRX1), prohibitin-2 (PHB2) and cardiolipin (CL). Parkin is an E3 ubiquitin ligase present in the cytoplasm (37), and PINK1 is a mitochondrial serine/threonine kinase. Studies have confirmed that PINK1 is an upstream regulatory protein of Parkin, and the two synergistically mediate the polyubiquitination process of damaged mitochondrial surface structures or functional proteins, and play a key role in depolarizing mitophagy degradation (38). Recent studies have shown that PINK1 can also induce mitophagy in a Parkin-independent manner. PINK1 recruits LC3 adaptor proteins OPTN and NDP52 to mitochondria, which in turn recruits autophagy initiator UNC-51-like kinase 1 (ULK1), double FYVE containing protein 1 (DFCP1), and WD repeat domain phosphoinositide-interacting protein 1 (WIPI1) to induce mitophagy (39). Additionally, synphilin-1 overexpression recruits PINK1, and the PINK-synphilin-1 complex can further recruit the E3 ubiquitin ligase SIAH-1 and promote the ubiquitination of mitochondrial proteins to induce mitophagy (40). NIX and BNIP3 are localized in the outer mitochondrial membrane (OMM) and belong to the BH3-only subfamily of B cell lymphoma-2 (Bcl-2) family proteins. NIX and BNIP3 can directly bind to LC3 through their BH3 domains to induce mitophagy (41, 42). Under hypoxic conditions, the mitochondrial outer membrane protein FUNDC1 induces mitophagy in a Parkin-independent manner by directly binding to LC3 (43). The activity of FUNDC1-induced mitophagy is regulated by phosphorylation and ubiquitination modifications (44, 45). BCL2L13 in the mammalian cell is a homologue of Atg32 in the yeast cell. Atg32 is the only mitophagy receptor molecule in yeast cells. BCL2L13 induces mitophagy through a Parkin-independent mechanism, and its mitophagy-inducing activity is regulated by phosphorylation modifications (46). FKBP8 is localized to the mitochondrial outer membrane and can bind to LC3A through its LIR amino acid sequence to mediate mitophagy, and this effect is independent of the PINK1/Parkin pathway (47). NLRX1 is a member of the nucleotide-binding oligomerization domain (NOD)-like receptor (NLR) family, and its N-terminus contains a mitochondrial localization sequence, making it the only NLR family mitochondrial protein (48). NLRX1 has been identified as a new mitophagy receptor involved in mitophagy (49), but its specific activation pathway and molecular mechanism have not been fully clarified. So far, the roles of many OMM proteins in mediating mitophagy have been confirmed, but the regulatory roles of inner mitochondrial membrane (IMM) proteins in mitophagy have not been fully elucidated. Recent studies have observed that PHB2 localized to the IMM is also a receptor protein that mediates mitophagy during stress and embryonic development (50). In addition, as a characteristic phospholipid of IMM, CL is involved in lipid-protein interactions and is one of the necessary raw materials for maintaining mitochondrial functions (eg, cristae formation, membrane fusion, etc.) (51). The study found that CL is a mitophagy receptor, and mitochondrial damage or membrane depolarization can cause CL to translocate from IMM to OMM, which is the initiation signal of CL-mediated mitophagy (52, 53).

### Regulatory mechanisms of mitochondrial biosynthesis

Mitochondrial biogenesis refers to the process of mitochondrial proliferation and mitochondrial system synthesis and individual synthesis in the life cycle of a cell (54). Mitochondrial biosynthesis is a self-protection mechanism initiated after tissue cells are attacked. Under the synergistic effect of mitochondrial genes and nuclear genes, mitochondrial biosynthesis maintains the stability of its own structure, function and quantity by synthesizing and replacing abnormal mitochondria, ensuring the normal metabolic process of cell mitochondria (55). Under physiological conditions, mitochondrial biosynthesis increases with cell proliferation, while under stress conditions such as low temperature, exercise, oxidative stress, and inflammation, mitochondrial biosynthesis is more pronounced, which can reduce the cell damage caused by ROS while ensuring the body’s extra energy demand (56). Therefore, mitochondrial biosynthesis can not only ensure the internal balance of mitochondria, but also enhance the cells’ anti-infection and anti-oxidation capabilities.

Mitochondrial biosynthesis includes the synthesis of the mitochondrial inner membrane, outer membrane and encoded proteins, the synthesis and transfer of nuclear gene-encoded proteins, and the replication of mitochondrial DNA (mt DNA) (57). This process is regulated by a variety of factors, among which peroxisome proliferator-activated receptor γ coactivator-1α (PGC-1α)/nuclear respiratory factors (NRFs)/mitochondrial transcription factor A (TFAM) signaling pathway is the most important pathway known to regulate mitochondrial biosynthesis. Overexpression of PGC-1α can increase the steady-state level of mt DNA, NRF-1/2 as a downstream transcription factor target of PGC-1α, is a major transcriptional regulator linking nuclear-encoded genes and mitochondrial biosynthesis, NRF-1/2 and PGC-1α bind and co-activates, while NRF-1/2 is involved in the expression of mt DNA-encoded mitochondrial proteins (58). TFAM is required for mt DNA transcription and replication, and binds to mitochondrial promoter sequences (including heavy and light chain specific promoters) in a sequence-specific manner, thereby efficiently activating mt DNA transcription. In conclusion, once PGC-1α is activated by the upstream adenosine 5’monophosphate-activated protein kinase (AMPK) and deacetylase through phosphorylation and deacetylation, it can bind to and help activate the downstream NRF-1/2, and then bind to the TFAM promoter to activate TFAM, complete and promote the replication and transcription of mt DNA for mitochondrial biosynthesis (59).

## Pharmacological effects of berberine targeting the mitochondria

**Figure 2:**
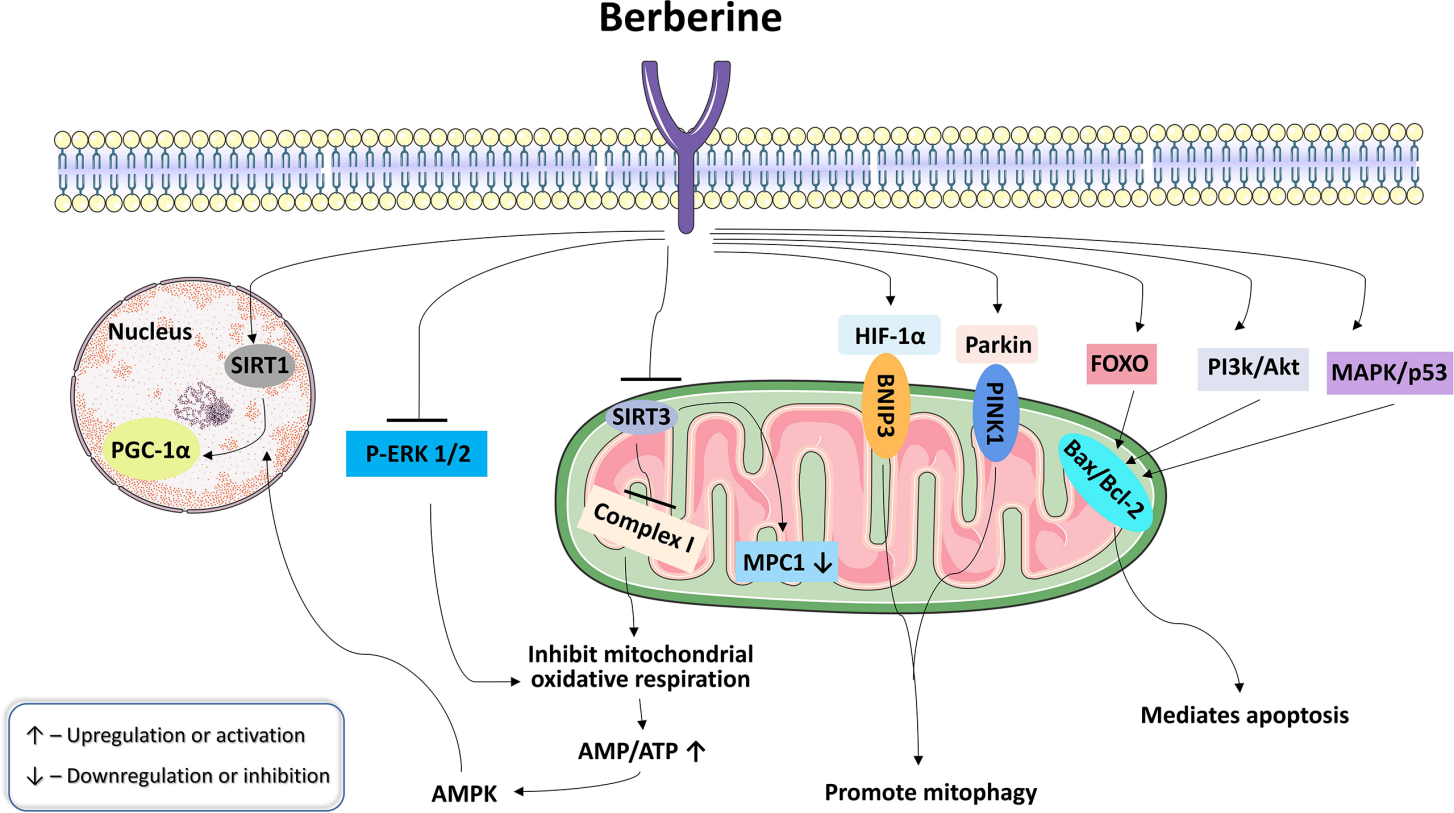
Pharmacological effects of berberine targeting the mitochondria.

The targeting mechanism of berberine exerting its pharmacological effects is complex. Previous studies have found that the regulation of mitochondrial activity is one of the ways that berberine exerts its pharmacological action (**Figure 2**, **Table 1**). Exploring the mechanism of berberine targeting the mitochondria has a certain guiding significance for promoting the clinical application of berberine.

**Table 1:** Research progress on the pharmacological effects of berberine targeting mitochondria.

	Effect	Experimental Model	Activities related to mitochondria	Ref.
**Glycolipid metabolism disorder**	Promote glycolysis	Human hepatoma cell line HepG2 and mouse skeletal myoblast C2C12	Inhibit mitochondrial respiratory chain complex I, suppress ATP synthesis and enhance glycolysis.	(60)
	Improve insulin sensitivity	LKB1^−/−^ mouse embryonic fibroblasts and L6 myotubes, HFD-induced C57Bl/6J mice	Inhibit mitochondrial respiratory chain complex I, activate AMPK and improve systemic insulin sensitivity.	(61)
	Inhibit gluconeogenesis	Primary hepatocytes from adult mice, ICR mice	Inhibit SIRT3, induce mitochondrial dysfunction, increase AMPK-related glucose uptake, block glucagon signaling and degrade its downstream target PEPCK1, thereby inhibiting gluconeogenesis. Preserve MPC1 acetylation by SIRT3 inhibition, and block mitochondrial pyruvate supply for gluconeogenesis due to MPC1 protein degradation.	(62, 63)
	Promote secretion of GLP-1	DIO mice	Inhibit ATP overproduction, control mitochondrial stress, protect intestinal L cells and promote GLP-1 secretion.	(64)
	Improve lipid metabolism	db/db mice, HFD-induced KO mice, C2C12 myoblasts	Upregulate the mitochondrial content in brown and white adipocytes, stimulate UCP1-mediated thermogenesis, and accelerate fat catabolism. Alleviate HFD-induced inhibition of mitochondrial β-OX through SIRT3-mediated LCAD deacetylation, and improve nonalcoholic fatty liver disease. Promote mitochondrial biosynthesis, improve fatty acid oxidation in an AMPK/PGC-1α-dependent manner and decrease abnormal ectopic lipid deposition in skeletal muscle.	(65–67)
	Others	HFD-induced Sprague Dawley rats, conditionally immortalized mouse podocyte, db/db mice	Improve mitochondrial biosynthesis and function *via* SIRT1-mediated mechanism. Activate the PGC-1α signaling pathway, and promote mitochondrial energy homeostasis and fatty acid oxidation. Inhibit the expression and translocation of Drp1, improve the fragmentation and dysfunction of mitochondria and reverse glycolipid metabolism disorder.	(68–70)
**Antitumor**	Induce tumor cell apoptosis	Pancreatic cancer cells, glioma cells, thyroid carcinoma cells, HepG2 cells, EBV-transformed B cells	Induce mitochondrial damage, target and regulate citrate metabolism and membrane transport in mitochondria, interfere with the synthesis and expression of downstream fatty acids and promote cancer cells apoptosis. Inhibit ERK activity, induce mitochondrial dysfunction, and lead to ATP depletion. Induce mitochondrial apoptosis, G0/G1 cell cycle arrest and suppress migration *via* PI3K-AKT and MAPK signaling pathways. Increase the production of ROS, activate the MAPK-p53 signaling pathway, and disrupt mitochondrial potential. Up-regulate the expression of FoxO1 and FoxO3, heighten BH3-only protein Bim expression, alter Bax/Bcl-2 ratio and induce mitochondrial dysfunction.	(71–75)
	Inhibit tumor cell proliferation	HepG2 xenograft model (CD1-nude mice were injected with HepG2 cells at a concentration of 10^6^ cells per mouse)	Decrease mtDNA copy number of tumor cells and inhibit cell proliferation.	(76)
**Protect cardiovascular** **system**	Improve myocardial ischemia/reperfusion injury	H9C2 cardiomyocytes	Activate HIF-1α/BNIP3 or PINK1-Parkin signaling pathway, promote mitophagy, reduce mitochondrial dysfunction, and improve myocardial I/R injury.	(77, 78)
	Inhibit cardiomyocyte apoptosis	Neonatal rat cardiomyocytes, Sprague-Dawley rats	Protect mitochondria (reduce mitochondrial membrane potential), increase the ratio of AMP to ATP and inhibit AMPK phosphorylation, elevate Bcl-2 expression, and suppress DOX-induced cardiomyocyte apoptosis.	(79)
	Improve cardiomyocyte hypertrophy	H9C2 cells	Activate the AMPK signaling pathway to inhibit mitochondrial fission, upregulate PGC1α to stimulate mitochondrial biosynthesis, restore autophagic flux disturbance, and prevent high glucose-induced cardiomyocyte hypertrophy.	(80)
	Others	C57BL/6J mice	Activate mitophagy *via* the PINK1/Parkin pathway and protect cardiac function in pressure overload-induced heart failure.	(81)
**Other effects**	Anti‐inflammatory	Macrophages infected with PR8 influenza virus	Induce mitophagy, decrease mitochondrial ROS, and suppress influenza virus-triggered NLRP3 inflammasome activation in macrophages.	(82)
	Protect nervous system	PC-12 cells, primary hippocampal neuron, dorsal root ganglion neurons isolated from STZ-induced diabetic rats	Inhibit oxidative stress and mitochondria dysfunction, and attenuate the cytotoxicity induced by tert-butyl hydroperoxide. Preserve the mitochondrial membrane potential and ATP generation, increase axonal mitochondrial density and length, improve mitochondrial motility and trafficking, and ultimately prevent synaptic loss. Activate AMPK in peripheral neurons and neuronal cells, promote PGC-1α mediated mitochondrial biogenesis, and augment autophagy to promote mitochondrial function.	(83–85)
	/	Human renal proximal tubular cell line HK-2, HFD-induced Sprague–Dawley rats, STZ-induced Wistar rats	Inhibit mitochondrial stress and endoplasmic reticulum stress pathways, and protect hypoxia/reoxygenation-induced apoptosis in human kidney proximal tubular cells. Upregulate mitochondrial SIRT3 activity, and reverse liver mitochondrial dysfunction in high-fat-fed rats. Inhibit the ROCK pathway, increase mitochondrial membrane potential, reduce ROS levels, and improve the cognitive impairment caused by diabetic encephalopathy.	(86–88)

### Regulate glycolipid metabolism

#### The relationship between mitochondria and the development of glycolipid metabolism disorder

Mitochondria, as the hub of body energy metabolism, are closely related to the occurrence and development of glycolipid metabolism disorder. Oxidative damage caused by mitochondrial ROS production plays a key role in the pathogenesis of glycolipid metabolism disorder, and is also an important pathophysiological feature of glycolipid metabolism disorder. In the respiratory electron transport chain, an appropriate amount of ROS can promote the secretion of insulin from islet β cells. However, patients with glycolipid metabolism disorder are exposed to a chronic state of hyperglycemia, causing oxidative stress damage to β cells and inhibiting β cell function and survival. Fluctuations in mitochondrial transmembrane potential can cause changes in intracellular adenosine triphosphate (ATP)/adenosine diphosphate (ADP) ratio and affect insulin secretion. Uncoupling protein 2 (UCP2), which is located in the inner mitochondrial membrane, is the master mitochondrial regulator of insulin secretion, and can cause proton leakage, decreased mitochondrial transmembrane potential, uncoupling of oxidative phosphorylation, and decreased ATP production, ultimately leading to a decrease in insulin secretion by β cells (89). Reduced mitochondrial biosynthesis is closely related to insulin resistance. Studies have shown that reduced mitochondria number and volume, and impaired mitochondrial respiration are found in muscle tissue in animal models of type 2 diabetes, obesity, and insulin resistance. The impaired mitochondrial respiration is mainly due to the downregulation of mitochondrial oxidative phosphorylation protein expression, resulting in mitochondrial biosynthesis reduced (90). The research of transgenic animal experiments indicated that overexpression of PGC-1α, which regulates mitochondrial biosynthesis, resulted in increased oxidative phosphorylation protein expression in rat muscle tissue and palmitic acid oxidation, enhanced insulin sensitivity, increased insulin-mediated glucose uptake, and decreased ROS production and inflammatory signaling (91). Activation of autophagy can induce islet β cells to be protected from oxidative stress, remove damaged organelle and their metabolites, promote mitochondrial and endoplasmic reticulum renewal, and maintain cellular homeostasis, whereas impaired autophagy will lead to mitochondrial dysfunction and endoplasmic reticulum stress, promote the occurrence and development of insulin resistance. Mitochondrial gene variation can also lead to glycolipid metabolism disorder. It may be related to insulin resistance, insufficient insulin secretion, oxidative phosphorylation damage, β cell apoptosis, and oxidative stress (92). The specific mechanism is not fully understood, further research and verification are needed. In addition, silent mating type information regulation 2 homolog 3 (SIRT3) located in the mitochondrial matrix plays a key role in energy metabolism, and its decreased activity can lead to impaired mitochondrial function and disturbance of body energy metabolism. SIRT3, which is considered to be a new potential target for regulating glycolipid metabolism disorder, can restore the activity of a series of metabolic enzymes in mitochondria through deacetylation, such as isocitrate, manganese superoxide dismutase and reduced coenzyme NADH, and increase the reduced glutathione content in mitochondria, reduce ROS generation, inhibit oxidative stress, and improve mitochondrial function (93, 94).

Mitochondrial damage plays an important role in the pathogenesis of glycolipid metabolism disorder, and mitochondrial dysfunction and glycolipid metabolism disorder have mutually causal relationships, forming a vicious circle. Mitochondrial membrane potential hyperpolarization, abnormal expression of uncoupling protein 2, reduced mitochondrial biosynthesis, and mitochondrial autophagy impairment lead to impaired mitochondrial function or structure, resulting in excessive production of ROS and aggravating insulin resistance. At the same time, mitochondrial gene variation is associated with the susceptibility to glycolipid metabolism disorder. Therefore, regulating mitochondrial biosynthesis and mitophagy, improving mitochondrial function, and mitochondrial gene therapy might be effective strategies to improve insulin resistance and prevent or delay glycolipid metabolism disorder.

#### Research progress of berberine in regulating glycolipid metabolism disorder by targeting the mitochondria

As the core mechanism of metabolic diseases such as diabetes, obesity and dyslipidemia, glycolipid metabolism disorder is quietly threatening human health. Previous studies have found that berberine can increase the expression of insulin receptors, promote glycolysis, enhance insulin sensitivity, promote the release and secretion of insulin, increase the consumption of glucose by liver cells, inhibit the differentiation of adipocytes, and regulate gut microbiota to regulate glycolipid metabolism, reduce triglyceride, cholesterol, low-density lipoprotein and blood glucose levels (95–97).

Mitochondria are essential for maintaining energy metabolism. A large amount of literature have reported that berberine inhibits hepatic gluconeogenesis, promotes the secretion of glucagon-like peptide-1 (GLP-1), upregulates glycolysis, enhances insulin sensitivity, and improves fatty acid oxidation by regulating mitochondrial activity, thereby regulating glycolipid metabolism. Xu et al. reported that elevated glycolysis may be a primary cause increased glucose consumption in hepatocytes and myotubes by berberine. *In vitro* experiment proved that berberine inhibited mitochondrial respiratory chain complex I, which led to the suppression of ATP synthesis, the enhancement of glycolysis, and the promotion of glucose metabolism, a process independent of AMPK activation (60). The results of animal experiments showed that berberine upregulates the mitochondrial content in brown and white adipocytes in db/db mice, and stimulates uncoupling protein 1 (UCP1)-mediated thermogenesis, thereby accelerating fat catabolism and improving obesity and other abnormal lipid metabolism diseases (65). Gomes et al. found that berberine improved mitochondrial biosynthesis and function *via* a silent mating type information regulation 2 homolog 1 (SIRT1)-mediated mechanism, thereby protecting against the deleterious effects of high-fat diet (HFD) feeding, hyperglycemia and fatty acids (68). Early studies found that berberine has direct inhibition of mitochondrial respiratory chain complex I, inhibiting mitochondrial oxidative respiration, increasing intracellular adenosine monophosphate (AMP)/ATP, thereby activating AMPK and improving systemic insulin sensitivity (61). Zhang et al. found that berberine inhibited mitochondrial function by inhibiting SIRT3. SIRT3 is a soluble protein deacetylase located in the mitochondrial matrix. It can interact with the subunits of the mitochondrial respiration Complex I. SIRT3 regulates the function of Complex I in the electron transport chain and maintains basal ATP yield. Inhibition of SIRT3 by berberine leads to mitochondrial dysfunction and increases the ratio of AMP/ATP, resulting in activating AMPK signaling, increasing AMPK-related glucose uptake, blocking glucagon signaling and degrading its downstream target PEPCK1, thereby inhibiting gluconeogenesis (62). Li et al. revealed that berberine reduced fasting hyperglycemia by reducing mitochondrial pyruvate import *via* inhibiting MPC1 function in the setting of lipid overload. Mitochondrial pyruvate import *via* mitochondrial pyruvate carrier (MPC) is a central step in hepatic gluconeogenesis. Berberine reduced hepatic acetyl CoA accumulation by limiting fatty acid oxidation and preserved MPC1 acetylation by SIRT3 inhibition, eventually blocking mitochondrial pyruvate supply for gluconeogenesis due to MPC1 protein degradation (63). However, recent studies identified that berberine can also regulate lipid metabolism by activating SIRT3. Inhibition of mitochondrial β-oxidation (β-OX) has been reported to be involved in the pathogenesis of the nonalcoholic fatty liver disease. SIRT3 regulates mitochondrial β-OX by deacetylating its substrate long-chain acyl-CoA dehydrogenase (LCAD). Xu et al. reported that berberine could alleviate HFD-induced inhibition of mitochondrial β-OX through SIRT3-mediated LCAD deacetylation, thereby improving nonalcoholic fatty liver disease in mice (66). Sun’s study showed that berberine controlled mitochondrial stress by inhibiting ATP overproduction in diet-induced obesity mice, thereby protecting intestinal L cells and promoting GLP-1 secretion (64). Yao et al. explored the effect of berberine on skeletal muscle lipid deposition and mitochondrial function from *in vivo* and *in vitro* experiments, and found that berberine could significantly reduce the content of triglycerides in the gastrocnemius muscle of obese mice, and it could promote mitochondrial biosynthesis and decrease abnormal ectopic lipid deposition in skeletal muscle by improving fatty acid oxidation in an AMPK/PGC-1α-dependent manner (67). Qin et al. found that berberine ameliorated fatty acid oxidation and related metabolic disorders in podocytes of diabetic kidney disease (DKD) mice by promoting PGC-1α to regulate mitochondrial energy homeostasis (69). Berberine activated the PGC-1α signaling pathway, which promoted mitochondrial energy homeostasis and fatty acid oxidation in podocytes. Lipid accumulation, excessive generation of mitochondrial ROS, mitochondrial dysfunction, and deficient fatty acid oxidation in DKD mouse models and cultured podocytes were suppressed by berberine. In addition, through inhibiting the expression and translocation of Drp1, berberine potently improved the fragmentation and dysfunction of mitochondria in podocytes, reduced mesangial matrix expansion, glomerular basement membrane thickening, podocyte damage, albuminuria and metabolic abnormalities of DKD (70).

### Antitumor

#### The relationship between mitochondria and tumorigenesis

The occurrence and development of tumors is a complex biological process involving multiple genes and multiple signaling pathways. Compared with normal cells, tumor cells have the characteristics of infinite proliferation, abnormal energy metabolism, increased ROS, tissue infiltration and metastasis, and resistance to cell death (98). In recent years, people have gradually realized that mitochondrial energy metabolism, oxygen free radical homeostasis, calcium ion homeostasis, apoptosis and autophagy abnormalities, and impaired mitochondrial dynamics play important roles in tumorigenesis, and proposed a new strategy for tumor therapy targeting the mitochondria.

The energy metabolism of tumor cells is significantly different from that of normal cells, and their rapid proliferation requires a large amount of biological macromolecules such as carbohydrates, lipids, and proteins. Mitochondria are the most important organelles that regulate cell metabolism. Abnormal mitochondrial metabolism is closely related to the proliferation, metastasis and survival of tumor cells (99). Tumor cells rely mainly on aerobic glycolysis to generate ATP instead of more efficient mitochondrial oxidative phosphorylation (100). The main reason might be that the mitochondrial function of tumor cells is irreversibly damaged, and oxidative phosphorylation is inhibited. This is the famous Warburg effect, which is one of the unique metabolic characteristics of tumor cells. Glycolysis not only provides energy for the life activities of tumor cells, but its intermediate metabolites can also provide carbon precursors for the synthesis of biological macromolecules such as amino acids, fatty acids, and nucleotides, and provide raw materials for the anabolism of tumor cells. In addition, the lactic acid produced by the glycolysis of tumor cells is released to the outside of the cell, forming a local acidic microenvironment, destroying the components of the extracellular matrix, and further enhancing the ability of tumor invasion and metastasis (101). Damaged mitochondria can also lead to abnormally active lipid metabolism and amino acid metabolism in tumor cells, providing raw materials for tumor cell survival and rapid proliferation (102, 103). Significantly increased levels of ROS in tumor cells are another feature of tumor cell metabolism. ROS are involved in many processes such as tumor cell transformation, proliferation, survival, migration, invasion, metastasis, and angiogenesis, and are closely related to the biological characteristics of tumor cells (104, 105). Mitochondrial dysfunction can lead to abnormal mitochondrial ROS homeostasis, resulting in the instability of the cell genome, tumor suppressor gene mutations, finally causing tumorigenesis (106). Mitochondria are both the main site of intracellular ROS generation and the main target of ROS attack. In the process of tumorigenesis, because mt DNA is not protected by histones and lacks the corresponding damage repair system, it is easy to be damaged by ROS and cause gene mutation. Mutations in mt DNA encoding proteins related to the oxidative phosphorylation system directly lead to the abnormality of the mitochondrial oxidative phosphorylation system, generating more ROS, which in turn aggravates further damage to mitochondrial function and ultimately drives the transformation of tumor cells into cell types that rely primarily on glycolysis for energy (107, 108). Besides the endoplasmic reticulum, mitochondria are another important intracellular calcium reservoir. Mitochondria can regulate intracellular calcium homeostasis through multiple calcium transport systems present on their membranes. Abnormal mitochondrial calcium homeostasis is closely related to mitochondrial dysfunction and the occurrence and development of tumors, but its specific mechanism is still unclear and needs to be further explored (109, 110). Decreased apoptotic capacity is one of the hallmarks of tumors. Apoptosis mainly includes two representative pathways, the mitochondrial apoptosis pathway and the death receptor-mediated apoptosis pathway. Mitochondria act as the regulatory center of apoptotic activity, and when mitochondrial apoptosis is inhibited, it may lead to tumor cells acquiring the ability to resist cell death (111, 112). Therefore, targeting the mitochondria to induce tumor cell apoptosis can be one of the strategies for tumor therapy. Mitophagy is a key adaptive effect of tumor cell survival by eliminating damaged mitochondria, controlling ROS production, reducing the risk of cell carcinogenesis, and reducing apoptosis (113, 114). Autophagy has dual regulatory effects on cell survival and death, and abnormal mitophagy is closely related to tumorigenesis. In addition, the impaired mitochondrial dynamic can lead to mitochondrial dysfunction, resulting in cell energy metabolism, proliferation, apoptosis, and mitophagy abnormal. The fusion and fission of mitochondria can also lead to changes in mitochondrial localization, which in turn affects the migration and invasion of tumor cells. Studies have found that key molecules involved in the regulation of mitochondrial fission and fusion, such as Drp1, MFN1, and MFN2, are abnormally expressed in a variety of tumor tissues, and may be closely related to tumor progression (115). The ability of tumor cells to migrate and invade is closely related to their ability to form pseudopodia, and the dynamic changes of microfilaments and microtubules involved in pseudopodia formation require a large amount of ATP generation. Therefore, as the main organ of ATP production, mitochondrial function, quantity and distribution can affect the formation of pseudopodia, which in turn affects the migration and invasion ability of tumor cells (116).

#### Research progress of berberine in antitumor by targeting the mitochondria

Mitochondria play an important role in abnormal energy metabolism, changes in ROS homeostasis, tissue infiltration and metastasis, and resistance to cell death in tumors. In recent years, with the in-depth study of the pharmacological mechanism of berberine, it has been found that berberine mainly achieves antitumor effects by interfering with tumor cell proliferation, inhibiting tumor cell invasion and metastasis, and inducing tumor cell apoptosis (117–119). At present, extensive *in vitro* experiments revealed that berberine can induce tumor cell apoptosis and inhibit tumor cell proliferation by regulating mitochondrial activity, thereby exerting antitumor pharmacological effects. Liu et al. revealed through metabolomics and transcriptomic studies that berberine can induce mitochondrial damage in pancreatic cancer cells, and may directly or indirectly interfere with the synthesis and expression of downstream fatty acids by targeting and regulating citrate metabolism and membrane transport in mitochondria, thereby inhibiting the growth of pancreatic cancer cells, promoting their apoptosis and inhibiting their invasion and metastasis (71). Sun et al. found that berberine-induced oncosis of glioma cells was correlated with ATP depletion *via* the mitochondrial dysfunction induced by the inhibition of extracellular regulated protein kinases (ERK) activity. The down-regulation of mitochondrial phosphorylated extracellular regulated protein kinases (p-ERK) by berberine inhibited aerobic respiration and led to glycolysis, an inefficient energy production pathway (72). Prior studies have established that berberine could inhibit thyroid carcinoma cells by inducing mitochondrial apoptosis, G0/G1 cell cycle arrest and suppressing migration *via* phosphatidyl inositol 3-kinase (PI3K)-protein kinase B (AKT) and mitogen-activated protein kinase (MAPK) signaling pathways (73). The mechanism of action of berberine in normal cells and tumor cells is similar such as regulating ROS. Oxidative stress plays an important role in the pathogenesis of many diseases. The beneficial effect of berberine is presumed to reside mostly in its antioxidant role (120). The mechanisms involved in the antioxidant role of berberine include scavenging ROS/RNS, removing free oxygen, reducing the destructiveness of superoxide ions and nitric oxide and so on. However, in tumor cells, berberine can increase the production of ROS and lead to the activation of the MAPK-p53 signaling pathway. The activated p53 and its downstream targets XAF1 and GADD45α interacted with PUMA, Bax and Bim in mitochondria, disrupted mitochondrial potential, and induced apoptosis in EBV^+^ B-lymphoma cells (74). Berberine can also induce mitochondrial dysfunction by significantly upregulating the expression of FoxO1 and FoxO3, thereby causing tumor cell apoptosis. Berberine significantly upregulated the mRNA expression of both FoxO1 and FoxO3a (75). FoxO transcription factors effectively heightened BH3-only protein Bim expression, and altered Bax/Bcl-2 ratio, culminating in mitochondrial dysfunction, caspases activation, and DNA fragmentation. Yan et al. found that berberine inhibited tumor growth and decreased mtDNA copy number of tumor cells in a dose-dependent manner in the HepG2 xenograft model, inhibiting cell proliferation (76).

### Protect the cardiovascular system

#### The relationship between mitochondria and the development of cardiovascular disease

Cardiomyocyte mitochondria are the main site of cardiac energy metabolism, and their dysfunction can lead to a variety of cardiovascular diseases. Mitochondrial quality control mainly ensures the relative stability of mitochondrial morphology, quantity and quality by regulating mitochondrial biogenesis, fusion, fission and autophagy to maintain the integrity of its structure and function. Currently, numerous studies have confirmed that mitochondrial quality control plays an important role in cardiovascular diseases such as ischemic heart disease, heart failure, atherosclerosis, diabetic cardiomyopathy (DCM) and hypertension. Therefore, mitochondrial quality control in the early stage of disease and reducing mitochondrial dysfunction have become new targets for cardiovascular disease treatment.

Mitochondrial biogenesis directly affects mitochondrial quality control and further affects myocardial functional status. Mitochondrial biogenesis disorder is an early change in the development of heart failure. Cardiomyocytes are sites of high energy consumption, and improving mitochondrial biogenesis disorder has cardioprotective effects (121). Even though genetic and environmental factors are different, mitochondrial dynamics imbalance can still be observed in a variety of cardiovascular diseases, such as excessive mitochondrial fission or reduced mitochondrial fusion involved in the progression of myocardial ischemia/reperfusion (I/R) injury and atherosclerosis, suggesting the balance between mitochondrial fusion and fission plays a significant role in the normal heart development and function (122, 123). Therefore, mitochondrial fusion and fission proteins may provide new therapeutic targets for a variety of cardiovascular diseases. Under normal circumstances, mitophagy can maintain the stability of mitochondrial function in cells, thereby ensuring normal heart functioning, whereas abnormal mitophagy is closely related to a variety of cardiovascular diseases. Cardiomyocyte mitophagy is enhanced under various cardiovascular stresses, and whether mitophagy is a potential protective mechanism or a pathological mechanism leading to cell death or further disease development remains inconclusive. Some studies have reported that mitophagy has a protective effect on ischemic myocardium (124), but other studies have shown that mitophagy is one of the causes of myocardial I/R injury (125). Abnormality of the mitochondrial quality control system will lead to mitochondrial dysfunction. Growing research has proved that mitochondrial dysfunction is the cause of many cardiovascular diseases. Mitochondrial dysfunction plays an important role in the process of atherosclerosis by affecting normal lipid metabolism, increasing ROS production, stimulating inflammatory responses and vessel wall remodeling (126, 127). Mitochondrial energy metabolism disorder is an important factor in causing myocardial I/R injury. The main mechanisms include reduced mitochondrial ATP production and excessive ROS production, resulting in oxidative stress, Ca^2+^ overload and persistent mitochondrial PTP opening (128). Increased ROS, decreased ATP production, Ca^2+^ overload, and inhibition of mitochondrial electron transport chain caused by mitochondrial dysfunction play an important role in the occurrence and development of hypertension (129, 130). Mitochondrial energy metabolism disorder not only reduces mitochondrial ATP synthesis but also increases mitochondrial ROS generation, thus playing an important role in the occurrence and development of heart failure (131). The pathogenesis of DCM is complex. Hyperglycemia, lipid accumulation, oxidative stress, inflammation, myocardial fibrosis, cardiomyocyte apoptosis, and mitochondrial damage are all involved in the pathophysiological process of DCM. Among them, mitochondrial damage caused by hyperglycemia leads to abnormal mitochondrial energy metabolism, increased ROS production, initiation of mitochondria-dependent apoptosis pathways, mitochondrial fission and fusion disorders, and changes in the content and structure of cardiolipin play a key role in the occurrence and development of DCM (132–134).

#### Research progress of berberine in protecting the cardiovascular system by targeting the mitochondria

Cardiovascular disease has become one of the diseases with the highest morbidity and mortality, and has received much attention in the world health system. With the continuous advancement of traditional Chinese medicine, a growing body of research has shown that traditional Chinese medicine has unique advantages in cardiovascular prevention and treatment. Berberine, as an active ingredient of the traditional Chinese medicine *Rhizoma Coptidis*, can regulate cardiovascular diseases such as hypertension, atherosclerosis, heart failure, DCM, ischemic heart disease, and arrhythmia by protecting vascular endothelial function, inhibiting inflammatory response, anti-platelet aggregation, dilating blood vessels, regulating autophagy, and anti-oxidation (135, 136).

Mitochondria are important targets for the treatment of cardiovascular disease. In recent years, with the deepening of the research on the pharmacological mechanism of berberine, it has been noticed that berberine can improve myocardial I/R injury, inhibit myocardial cell apoptosis, and protect cardiac function by regulating mitochondrial activity. *In vitro* experiments show that berberine could not only upregulate PTEN-induced putative kinase 1 (PINK1) and Parkin levels, activate the PINK1-Parkin signaling pathway, promote mitophagy, induce autophagic flux, reduce mitochondrial dysfunction, and improve myocardial I/R injury, but also can promote mitophagy, reduce myocardial enzyme activity, induce cardiomyocyte proliferation, inhibit cardiomyocyte apoptosis, and protect the heart from myocardial I/R injury, possibly through the hypoxia-inducible factor 1α (HIF-1α)/BNIP3 pathway (77, 78). Studies have shown that doxorubicin (DOX) could rapidly induce mitochondrial damage and increase intracellular AMP/ATP ratio, which in turn activates AMPK phosphorylation and promotes cardiomyocyte apoptosis. Lv et al. reported that berberine suppressed DOX-induced cardiomyocyte apoptosis *via* protecting mitochondria (reducing DOX-induced loss of mitochondrial membrane potential), reducing the increased ratio of AMP to ATP and inhibiting AMPK phosphorylation as well as elevating Bcl-2 expression in the early stage of DOX treatment (79). Hang et al. proposed that berberine could prevent high glucose-induced cardiomyocyte hypertrophy by activating the AMPK signaling pathway to inhibit mitochondrial fission, upregulating proliferator-activated receptor-gamma coactivator 1α (PGC1α) to stimulate mitochondrial biosynthesis, and restoring autophagic flux disturbance (80). Animal experiments confirmed that berberine activated mitophagy *via* the PINK1/Parkin pathway and protected cardiac function in pressure overload-induced heart failure (81).

### Other pharmacological effects

Berberine has a wide range of pharmacological effects and can be applied to various diseases. In addition to hypoglycemic, lipid-lowering, cardiovascular protection, and antitumor effects, berberine also has pharmacological activities such as anti-inflammatory, antibacterial, and protection of the central nervous system. In recent years, a large number of studies have shown that berberine targeting the mitochondria as therapeutic targets to regulate the structure and function of mitochondria, thereby exerting pharmacological effects. Yu et al. found that berberine pretreatment had a protective effect on hypoxia/reoxygenation-induced apoptosis in human kidney proximal tubular cells, and the mechanism was related to the inhibition of mitochondrial stress and endoplasmic reticulum stress pathways (86). Liu et al. reported that berberine suppresses influenza virus-triggered NOD-like receptor pyrin domain-containing protein 3 inflammasome activation in macrophages by inducing mitophagy and decreasing mitochondrial ROS (82). Li et al. found that berberine attenuated the cytotoxicity induced by tert-butyl hydroperoxide *via* inhibiting oxidative stress and mitochondria dysfunction in PC-12 cells (83). Berberine significantly suppressed cytochrome c expression, upregulated the ratio of Bcl-2/Bax, and ameliorated mitochondrial dysfunction by optimizing mitochondria membrane potential status and ATP production, playing a protective role in anti-apoptotic and anti-oxidative stress. Zhao et al. reported that berberine protected against Aβ-induced axonal mitochondrial abnormalities in primary cultured hippocampal neurons by preserving the mitochondrial membrane potential and ATP generation, increasing axonal mitochondrial density and length, and improving mitochondrial motility and trafficking, ultimately preventing synaptic loss (84). Previous research indicated that berberine activates AMPK in peripheral neurons and neuronal cells, which promotes PGC-1α mediated mitochondrial biogenesis, and augments autophagy to promote mitochondrial function (85). The improved mitochondrial function by berberine further strengthened redox homeostasis through stimulation of endogenous antioxidant systems and inhibition of neuroinflammation. These effects lead to increased nerve conduction velocity, improved nerve blood flow, and decreased hyperalgesia in diabetic neuropathy. Teodoro et al. found that mitochondria isolated from the livers of high-fat-fed rats exhibited decreased calcium accumulation capacity and impaired oxidative phosphorylation capacity, such as impaired mitochondrial membrane potential, oxygen consumption, and cellular ATP levels, whereas berberine could reverse liver mitochondrial dysfunction in high-fat-fed rats by upregulating mitochondrial SIRT3 activity (87). Tian et al. proposed that berberine improved the cognitive impairment caused by diabetic encephalopathy by inhibiting the Rho/Rho kinase (ROCK) pathway in diabetic encephalopathy rats, increasing mitochondrial membrane potential and reducing ROS levels (88).

## Summary and outlook

Mitochondria are ubiquitous organelles that mainly consist of a bilayer-membrane structure and matrix. It is the ‘power station’ of cells and plays a core role in cell energy production, metabolism and signal transmission. Notably, mitochondria are critical for respiration, apoptosis, and mitochondrial DNA inheritance, and alterations in their structural and functional integrity may lead to ATP depletion, ROS overproduction, decreased mitochondrial membrane potential, impaired mt DNA and other mitochondrial dysfunction, and further mediate cell apoptosis and inflammatory responses (137). The mitochondrial quality control system mainly includes mitochondrial biosynthesis, dynamics and autophagy. As an important endogenous mechanism for maintaining mitochondrial homeostasis in cells, the mitochondrial quality control system has two opposites. It not only involves the regeneration of mitochondria, but also includes the removal of damaged or aging mitochondria, which is the core of maintaining the normal mitochondrial structure and exerting the normal physiological function of mitochondria (138). Recent studies have suggested that mitochondria are the target organelles for many drugs and poisons. Berberine is a common active ingredient in a variety of heat-clearing and detoxifying traditional Chinese medicines. Its functions such as heat-clearing and detoxifying and antibacterial have been used in clinical practice, and it also has various pharmacological activities such as antitumor, hypoglycemic, blood lipid regulation, hypotensive and antiarrhythmic effects. With more in-depth research, the mechanism of berberine’s pharmacological activity by targeting the mitochondria has been elucidated. Berberine can affect the quality control and function of mitochondria to a certain extent by regulating the expression of oxidative stress, mitochondrial fusion and fission, mitophagy, mitochondrial biosynthesis, and intracellular calcium regulation, thereby exerting pharmacological effects.

In improving glycolipid metabolism disorder, berberine can directly inhibit mitochondrial respiratory chain complex I or regulate the function of mitochondrial respiratory chain complex I by inhibiting SIRT3 in the mitochondrial matrix, thereby inhibiting ATP synthesis, upregulating the ratio of AMP/ATP, activating the AMPK signaling pathway to inhibit hepatic glycogen production, promote glycolysis, and improve insulin sensitivity. Berberine can also improve mitochondrial dysfunction, regulate mitochondrial energy homeostasis, and then regulate glycolipid metabolism disorder by promoting the expression of PGC-1α and upregulating mitochondrial biosynthesis, inhibiting the expression and mitochondrial translocation of Drp1 and downregulating mitochondrial fission. In antitumor progression, berberine can induce tumor cell apoptosis by activating PI3K-AKT and MAPK-p53 signaling pathways or promoting the expression of FoxO1 and FoxO3, and upregulating pro-apoptotic proteins such as Bax and Bim. Berberine also exerts antitumor effect by inhibiting cell proliferation by inhibiting ERK activity and inducing mitochondrial dysfunction or reducing mtDNA copy number. In the prevention and treatment of cardiovascular diseases, berberine mainly protects cardiac function by activating the PINK1-Parkin, HIF-1α/BNIP3 signaling pathways and promoting mitophagy. In addition, berberine can also improve mitochondrial function by protecting mitochondrial membrane potential, activating mitochondrial apoptosis signaling pathway, and promoting mitochondrial biogenesis, thereby exerting pharmacological effects such as anti-inflammatory and nerve protection. In summary, berberine mainly regulates the function of the mitochondrial respiratory chain, activates the important energy homeostasis regulator AMPK, regulates glycolipid metabolism, regulates mitochondrial apoptosis, promotes tumor cell apoptosis, promotes mitophagy to reduce mitochondrial dysfunction and protect heart function.

Mitochondria are important targets for berberine to exert its pharmacological effects, but the regulatory mechanism of berberine on mitochondria still needs to be further studied. Firstly, there are differences in mitochondria and their functions in different species. Different concentrations and dosages of berberine affect the regulation of mitochondria. Therefore, in order to prevent differences in research results, the research design should be more standardized. Secondly, the relevant research is still at an exploratory stage, and the research types mainly are animal experiments or cell experiments, and lack of *in vitro* mitochondrial experiments or clinical trials to evaluate the mechanism of action. Most importantly, there is currently a lack of multi-target, multi-path, multi-dimensional, and in-depth systematic research. Current research mostly focuses on the regulation of a specific pathway, the detection indicators are relatively limited, and pharmacokinetics is lacking. In addition, mitochondrial quality control is a continuous dynamic process, and static assessment of the roles and mechanisms of independent components of the mitochondrial quality control system at a single time point may generate research bias. Therefore, it is necessary to further explore the influence mechanism on different signaling pathways and their related factors in the process of applying berberine to intervene in mitochondria in the treatment of diseases. The normal maintenance of cell activity and physiological function relies on the cooperation of multiple intracellular and extracellular molecules and signaling pathways, and mitochondria are extensively involved in the regulation of physiological activities. Consequently, we need to comprehensively explore the mechanism of berberine regulating mitochondria to exert pharmacological effects through different technical means and different signal transduction pathways. The application of emerging omics technologies and methods such as mitochondrial proteomics and epigenetics is expected to bring light to screen and discover the targets of berberine.

## Author contributions

JT developed the review question. The initial literature review was performed by XF. The first draft of the manuscript was written by XF with all authors commenting on subsequent versions of the manuscript. All authors read and approved the final manuscript.

## Funding

This work was supported by the National Natural Science Foundation of China (81904187), Capital Health Development Research Project (CD2020-4-4155), CACMS Outstanding Young Scientific and Technological Talents Program (ZZ13-YQ-026), CACMS Innovation Fund (CI2021A01601), Innovation Team and Talents Cultivation Program of National Administration of Traditional Chinese Medicine (ZYYCXTD-D-202001), Open Project of National Facility for Translational Medicine (TMSK-2021-407).

## Acknowledgments

We would like to thank all the authors for their contribution to the realization of this manuscript.

## Conflict of interest

The authors declare that the research was conducted in the absence of any commercial or financial relationships that could be construed as a potential conflict of interest.

## Publisher’s note

All claims expressed in this article are solely those of the authors and do not necessarily represent those of their affiliated organizations, or those of the publisher, the editors and the reviewers. Any product that may be evaluated in this article, or claim that may be made by its manufacturer, is not guaranteed or endorsed by the publisher.

## Glossary

**Table:**

ADP	adenosine diphosphate
AKT	protein kinase B
AMP	adenosine monophosphate
AMPK	adenosine 5’monophosphate-activated protein kinase
ATP	adenosine triphosphate
Bcl-2	BH3-only subfamily of B cell lymphoma-2
Bcl2L13	Bcl-2-like protein 13
BNIP3	Bcl-2/adenovirus E1B 19 kDa interacting protein 3
β-OX	β-oxidation
CL	cardiolipin
DCM	diabetic cardiomyopathy
DFCP1	double FYVE containing protein 1
DKD	diabetic kidney disease
DOX	doxorubicin
Drp1	dynamin-related protein1
ERK	extracellular regulated protein kinases
Fis1	fission protein 1
FKBP8	FK506 binding protein 8
FUNDC1	FUN14 domain-con-taining protein 1
GLP-1	glucagon-like peptide-1
hERG	human ether-a-go-go-related gene
HFD	high-fat diet
HIF-1α	hypoxia-inducible factor 1α
IMM	inner mitochondrial membrane
I/R	ischemia/reperfusion
LCAD	long-chain acyl-CoA dehydrogenase
LD_50_	median lethal dose
MAPK	mitogen-activated protein kinase
Mff	mitochondrial fission factor
Mfn1	mitofusin 1
Mfn2	mitofusin 2
MiD49	mitochondrial dynamics proteins of 49 kDa
MiD51	mitochondrial dynamics proteins of 51 kDa
MPC	mitochondrial pyruvate carrier
mt DNA	mitochondrial DNA
NIX	Nip3-like protein X
NLR	NOD-like receptor
NLRX1	nucleotide-binding domain and leucine-rich-repeat-containing proteins X1
NOD	nucleotide-binding oligomerization domain
NRFs	nuclear respiratory factors
OMM	outer mitochondrial membrane
OPA1	optic atrophy 1
p-ERK	phosphorylated extracellular regulated protein kinases
PGC-1α	peroxisome proliferator-activated receptor γ coactivator-1α
PHB2	prohibitin-2
PI3K	phosphatidyl inositol 3-kinase
PINK1	PTEN induced putative kinase 1
ROCK	Rho/Rho kinase
ROS	reactive oxygen species
SIRT1	silent mating type information regulation 2 homolog 1
TFAM	mitochondrial transcription factor A
UCP1	uncoupling protein 1
UCP2	uncoupling protein 2
ULK1	UNC-51-like kinase 1
WIPI1	WD repeat domain phosphoinositide-interacting protein 1
